# Adaptive coordination in surgical teams: an interview study

**DOI:** 10.1186/s12913-015-0792-5

**Published:** 2015-04-01

**Authors:** Jasmina Bogdanovic, Juliana Perry, Merlin Guggenheim, Tanja Manser

**Affiliations:** Industrial Psychology and Human Factors Group, Department of Psychology, University of Fribourg, Fribourg, Switzerland; Division of Plastic Surgery and Hand Surgery, University Hospital Zurich, Zurich, Switzerland; Institute of Patient Safety, University Hospital Bonn, Bonn, Germany; Department Management, Technology, and Economics, Zurich, ETH Switzerland

**Keywords:** Interview study, Adaptive coordination, Teamwork, Non-technical skills, Surgery, Patient safety

## Abstract

**Background:**

Effective teamwork has been recognised as a major contributor to safe patient care in surgery. Previous research has highlighted the importance of adaptive coordination for effective performance in acute care settings. Expanding this line of research this study explores the coordination behaviours and adaptive coordination strategies employed by surgical teams and identifies relevant situational characteristics influencing those coordination processes.

**Method:**

We conducted a qualitative content analysis of semi-structured interviews with 33 surgical team members (nurses and physicians) from different specialties and hospitals.

**Results:**

We identified coordination behaviours (i.e. task management, information management, teaching and leadership) and adaptive coordination strategies triggered by varying requirements due to non-routine events, intraoperative complications and differing level of experience among operating room staff. Interviewees highlighted the importance of effectively managing challenging moments and the supporting effect of positive climate on teamwork.

**Conclusions:**

This study complements previous research on the non-technical skills underpinning safe performance in surgical teams. It highlights the central role of coordination and points out the ways in which situational variability requires the team to behave adaptively.

**Electronic supplementary material:**

The online version of this article (doi:10.1186/s12913-015-0792-5) contains supplementary material, which is available to authorized users.

## Background

Over the last decade, the conceptualisation of the underpinnings of safe patient care has changed considerably [1]. Evidence from retrospective incident analyses and prospective observational studies indicates that many of the factors contributing to adverse events originate from teamwork failures rather than from a lack of clinical skills [2]. In surgery, teamwork and communication problems were shown to be a major contributor to surgical injuries [3-5]; second only to lack of competence [6]. Thus, the influence of teamwork on safe patient care has recently become a major focus of research, particularly in the operating room (OR) [2,7-10].

Previous studies on teamwork in surgical teams have focused mainly on the identification and rating of non-technical skills [11], i.e. the “cognitive, social and personal resource skills that complement technical skills, and contribute to safe and efficient task performance“ (p. 1) [12]. Interview studies aimed at eliciting these skills have frequently used the critical incident technique [13] as a method for examining expert performance relevant to safe care. However, the shift from routine practice to managing critical situations has so far been rather implicit in research on teamwork in surgical teams [2,9].

There is a growing consensus in the literature that behaviours helping to increase a team’s ability to adapt to variability, disturbance, etc. ensure high performance. Coordination, and more specifically, the adaptation of coordination strategies to the situational requirements (i.e. adaptive coordination) has been identified as a central mechanism of safe and effective performance in healthcare and other high-risk work environments [14-16].

Coordination can be defined as a “process by which team resources, activities, and responses are organized to ensure that tasks are integrated, synchronized, and completed within established temporal constraints” (p. 345) [17]. In surgical teams, effective coordination is essential because different team members routinely perform multiple interdependent tasks, simultaneously requiring them to exchange task related information or mutually adjust their actions. Due to the potential for situational variability (e.g. intraoperative complications) inherent to surgical practice, we assume that successful surgical teams will constantly adapt their coordination behaviour to the evolving situation.

Despite the importance of adaptive coordination documented in many teamwork contexts, including healthcare [18-21], studies investigating teamwork in surgery have not yet explicitly addressed the issue of (adaptive) coordination. In order to better understand effective teamwork in surgical teams, this interview study explored the following two research questions. Which coordination behaviours and adaptive coordination strategies do surgical teams employ to ensure effective task performance? Which situational characteristics do surgical team members perceive to influence team coordination (i.e. facilitate or hinder coordination, require adaptations)? This article reports on the perceptions of surgical team members on the specific coordination behaviours and the need for adaptive coordination in surgery. These findings can be used to develop descriptive observation systems needed to identify effective coordination behaviours and adaptive coordination strategies by linking them to performance data in future studies.

## Methods

### Study design

Semi-structured interviews with subject matter experts enable the interviewer to explore a specific topic while still providing flexibility for interviewees to elaborate on emerging aspects that were not explicitly asked about by the interviewer [22]. Since coordination behaviours and adaptive coordination strategies were not yet examined in the setting of surgical teams an explorative study design was chosen.

### Interview design

The interview schedule was designed using relevant topics from previous research regarding adaptive coordination in anaesthesia teams [15,16,20] whilst taking into account the wider literature on teamwork in surgery [8,23-25]. Interview questions focused on responsibilities and roles concerning clinical tasks and leadership, coordination behaviours as well as adaptation requirements and strategies (see Additional file 1). Interviewees were asked to refer to the perioperative phases of surgery.

### Participants

A total of 33 clinicians were included in this study. An overview of the demographic data is provided in Table 1. Most participants were from the Division of Plastic and Reconstructive Surgery of a major Swiss teaching hospital. Potential participants were informed about the study through a presentation to nurses and physicians at departmental meetings and flyers providing contact information. However, to prevent single site bias we recruited four surgeons of different specialties from two other Swiss hospitals using the same recruitment strategy.

**Table 1:** **Demographics of interview participants**

	**Surgeons**		**Nurses**	**Total**
	**Plastic and Reconstructive Surgery**	**Orthopaedic, Visceral and Neurosurgery**	**Plastic and Reconstructive Surgery**	
	**N = 13**	**N = 4**	**N = 16**	**N = 33**
Work experience in years	10.2 (±5.94)	17.6 (±2.51)	20.12 (±8.67)	15.9 (±8.57)
Current position in years	4 (±2.54)	5 (±2)	8.16 (±6.71)	6.1 (±5.29)
Age in years	36.5 (±4.88)	47.25 (±5.91)	51.85 (±8.78)	45.1 (±10.02)

Note: All values given as mean (±standard deviation).

### Procedure

Interviews took place between January and June 2012. Ethical approval was granted by the Cantonal ethics committee (KEK-ZH-Nr. 2011–0313). Participation was voluntary and informed consent was obtained from all participants. Interviews were conducted by two psychologists (JB and JP) familiar with the context in which surgical teams function. Individual interviews were carried out at locations and times convenient to the participants and lasted on average 40 minutes. Data were collected until saturation was reached (i.e. no new information was generated from additional interviews) [26]. In fact, in this study we applied this criterion twice. Once we did not obtain new information from participants in one hospital we sought input from interviewees from other hospitals and surgical specialties.

### Analysis

Qualitative content analysis [27] was performed using the MaxQDA Software [28]. The interview coding system (Table 2) was developed using a combined deductive and inductive approach [26]. In a first step we analysed five randomly selected transcripts a) using coordination categories previously established in healthcare teams (e.g. task management) [15,20,21] and b) allowing for additional themes to emerge from the data material (e.g. challenging moments). Following the steps of the qualitative content analysis [27] relevant data was extracted from the transcripts by identifying thematic text segments. Themes were then sorted into representative categories, the content was abstracted, the amount of data was further reduced and redundancies were deleted. The initial interview coding system was discussed and revised several times within the research team using the constant comparative method [26]. Subsequently, all transcripts including the five interviews used to develop the coding system were coded by one psychologist (JB) applying the interview coding system (Table 2). Ten percent of all interview segments identified in the data material were randomly selected and independently coded by a second psychologist (MD) to determine inter-rater agreement using Cohen’s Kappa statistics [29].

**Table 2:** **Coding system for interview analysis**

***Coordination behaviours and adaptive coordination strategies***		
Task management (k = .88)*	Planning	It’s more that you see, in patients who are at a certain age and have comorbidities, that you really consider how far you can go in a procedure, what their limitation is, as far as one can gauge this, with respect to operative time, with respect to blood loss, regarding pain level… So that you anticipate, so that that one defines clear parameters. [S12]
	Task distribution	Well, that someone does not possess the skills for the task at that moment. (…) That we swap with each other, so that the distribution of tasks works again and we can carry on. [N04]
	Prioritisation	It also depends on the importance because if there are problems with the patient, then of course I’m first going to call on one of the anaesthesia consultants, before I unpack any material. [N04]
	Delegation	Or I realise, I need something really urgently, then I know I can send someone outside, and then it will be brought. [N01]
	Clarification of task	For example, if there is nothing mentioned on the list of split-skin grafting, if you cannot quite close the wound, then I can ask, don’t you need split-skin, can you close the wound like this and so on. [N06]
	Assistance	The scrub nurse in the sense that when I realise that she can’t really reach over to the surgical field, that I lend her a hand so to speak. [S02]
	Team and process monitoring (incl. routine checks)	In the beginning, before the operation starts, there are checklists (…). We have also, in my opinion a very efficient and good checklist, it also gives a lot of security and confidence that you really think of things that are important. Everyone needs to be involved; the anaesthetist, the surgeon and the nurses. [N11]
Information management (k = .85)*	Procedure and patient related information	When intraoperatively some bullshit happens, when you cut into some vessel and then it bleeds or some such thing, any unforeseen events (…) that has to be communicated immediately. [S09]
	Situation assessment	I must have a willingness not to rush through this procedure as a lone fighter, but keep eyes and ears open, around me. When I hear in the background, the patient, the sound of the monitor there behind the green cloth is getting slower, there sits an inexperienced anaesthesia trainee. (…) This requires attention from all team members to the other team members, what do they need, what do they want. [S08]
	Team member information	I need to know basically what the other person knows. If I do not know (…) I have to check. [N05]
	Decision making	If I notice surgically for example, that’s not what we expected, then you have to discuss the plan anew (…) and that is only possible as a team. This requires communication to come to a joint decision on the onward progress of the operation. I think it is wrong for one to then decide alone. [S08]
Teaching (k = .94)*	Explanation/guidance	There may be an intern for example, who is allowed to do something in the OR, for example sew up a wound, and he doesn’t do terribly well or has not often sewn, then you can teach him or help him. [S10]
	Balancing teaching and other tasks	But there are those [procedures] that are more difficult, or the doctors are nervous, or it bleeds, then you have really no time, you have to concentrate on the operation. (…) Then you cannot look after anyone in addition. But most often this phase is over quickly, and then you can concentrate on the trainee on top of everything. [N07]
Leadership (k = 1)*	Leadership role	But I think it’s a co-dominance there, because everyone has his own specialty and for his specialty the ultimate authority. [S02]
	Change of leader	That perhaps the surgeon has the lead, but the anaesthetist says, hey, now you have to stop the patient is in pain, you have to wait now. [N07]
***Situational and contextual drivers***		
Challenging moments (k = .71)*	Unexpected situations	If it’s a big case, then the surgeons can choose a completely different approach intraoperatively. Simply because vessels are not such as one had hoped, or because it bleeds more or because there is an infection, you have not seen from the outside, that can alter a procedural step considerably. [N13]
	Anticipated challenges	If an intricate step of the operation (…) where they have sewn small vessels under the microscope, which simply must be done well, quickly and cleanly, which is a step where everyone knows this is not the time to just ask some questions about something else, it takes maximum concentration. [S14]
Climate (k = 1)*	Communication openness/work atmosphere	But the nursing assistant, I find, also has the right, if she sees something, if what I do is not good, that she tells me that, that’s something I expect. [N16]

*Inter-rater agreement given in Cohen’s Kappa (k) separately for each main category.

## Results

Overall inter-rater reliability on the main and subcategory levels of the interview coding system was excellent (Cohen’s Kappa k = .87 and k = .83, respectively) [29]. Inter-rater reliability for each main category is listed separately in Table 2.

The themes emerging from the interviews fell into two main groups: a) *coordination behaviours and adaptive coordination strategies* and b) *situational and contextual drivers*, including the team’s management of *challenging moments* and the facilitating effect of a positive *climate* (i.e. communication openness, work atmosphere) on teamwork.

### Coordination behaviours and adaptive coordination strategies

#### Task management

The coordination behaviours and adaptive coordination strategies summarised under task management refer to *planning*, *task distribution, prioritisation, delegation, clarification of task, team and process monitoring* and *assistance*.

*Planning* is concerned with discussion within the whole surgical team or between certain team members regarding surgical procedure and any peculiarities (e.g. supplementary instruments, time management, patient specifics). It takes place predominantly before the day of surgery with different team members being involved in different topics. If possible, deviations from standards are communicated before entering the OR.

*Prior to surgery. This is for me; each team member must know beforehand what he has to do. Only then can he fulfil his role effectively and correctly. When we disagree, we need to work out a solution. One can work this out in advance so that everyone knows what he has to do. This is more effective and safer* [S08].

Finally, the whole team conducts the preoperative checklist right before the surgery starts to ensure that everyone is aware of the plan and the anticipated challenges.

During surgery the preoperatively discussed plan serves as a shared mental model [30,31] or template against which intraoperative findings and events can be contrasted and potential adaptations discussed. With this in mind, situational decisions are being communicated and critical steps and their specific requirements are often announced to ensure synchronised activity of the whole team. This was described as occurring more frequently with increased complexity of the surgical procedure.

*If the microsurgical part comes you have to prepare a microscope or whatever. And also with anaesthesia, (…) there comes a point where I need high blood pressure or relaxation or not, these are the things you can determine only during surgery and that you must or want to communicate* [S03].

Due to the fact that this study was conducted at a teaching hospital, participants mentioned that during surgery there is an intensive exchange between the operating surgical trainee and the supervising consultant surgeon. The consultant surgeon expects the trainee to verbalise the planned surgical steps but often with decreasing frequency as the trainee gains experience. This explicit verbalisation of the planned course also serves to synchronise mental models of the consultant and the trainee. Participants highlighted that intraoperative planning including task *prioritisation* relies on maintaining situation awareness [32] through close monitoring of the procedure and the overall team. Clinicians evaluate the situation to make intraoperative decisions and to point out to their colleagues what has to be prioritised under the current circumstances (see also situation assessment and decision making).

Thus, throughout surgery deviations from standards or previously established plans can function as triggers to adapt the usual coordination strategy. Interviewees also mentioned complications (e.g. anaesthetic problems) and incidental findings (e.g. inflammations) as triggers to adapt their coordination behaviours. Such unexpected events will increase the team’s communication about task management issues in terms of priorities, pacing and team composition. For example, in case of deteriorating patient condition, tasks to stabilise the patient have to be prioritised, surgery may have to be interrupted and the team may call a more experienced clinician for help. Moreover, incidental findings often lead to strategic changes of the primary surgical plan. If alternatives have been discussed before, the team may switch plans more easily. If not, the surgeon may interrupt the procedure to discuss further steps with the team or to get advice from a more experienced colleague.

The general *task distribution* was also described to take place predominantly before the surgical procedure. The consultant surgeon decides which tasks are assigned to whom within the team of surgeons. Nurses usually distribute the tasks between themselves. Criteria for task assignment are the level of training, experience and competence as well as availability of staff. Tasks are often assigned to certain team members to enable teaching. Intraoperatively the general task distribution is constantly being assessed and changed if necessary to ensure that it is still appropriate for the current situation. As one surgical trainee stated:

*Actually, this is already clarified before the surgery, but if new issues emerge, we have to make this explicit again during the operation. For example, if difficulties occur somehow, that the senior physician says, I’m now taking over to do this step* [S11].

Reasons for such adaptations can be, for example, deviation from the planned procedure, lack of experience or competence, illness, staff shortages, or mental block:

*Once, a trainee called me and said, I’ve cut the wrong finger (…). I then took over the operation, because he was so agitated, totally lost (…) That was such a psychological burden for him that he could proceed no further* [S03].

Besides function-specific *delegation* which is usually determined by profession (e.g. surgeon asks the scrub nurse to hand over a certain instrument), additional tasks, which can be fulfilled by any team member and are not specifically assigned beforehand, are delegated on an ad-hoc basis throughout surgery and depend on situational occurrences. Team members will also take over tasks of their colleagues to balance the workload within the team (e.g. the anaesthetist might help the scrub nurse if the circulating nurse is busy with another important task). These minor adjustments to the general task distribution and the temporary assistance across the boundaries of professional roles create situational flexibility, minimise pressure and enable a smoothly running procedure.

This dynamic delegation of tasks is closely related to *assistance* that team members can offer or ask for during the procedure. Interviewees mentioned that they usually appreciate assistance in form of “thinking ahead”, anticipating their needs and giving hints. Frequent examples were passing or readjusting equipment, helping with re-positioning of the patient or offering a chair to the surgeon. In case that a certain task is not clearly communicated participants mentioned to ask *clarifying questions* to avoid mistakes.

*Team and process monitoring* is a theme that newly emerged from the data material. It was described as an important coordination behaviour that involves routine checks as well as a continuous monitoring as the situation unfolds. In terms of routine checks, all participants mentioned that they always use the WHO surgical safety checklist [33] before and after every surgical procedure. They described the checklist as a very important moment for coordination of the whole team. During surgery, team members monitor the activities of other team members, particularly those with less experience, in accordance with their responsibility for certain tasks and work areas.

#### Information management

The coordination behaviours and adaptive strategies that can be summarised under information management refer to *procedure and patient related information*, *situation assessment*, *team member information* and *decision making*.

Participants mentioned that during surgery team members are continuously exchanging *procedure and patient related information*. Anaesthetists and surgeons routinely exchange information regarding patient condition, drug administration and length of surgical procedure. Nurses and surgeons exchange information about supplementary material and instruments. If there are two surgical sites at a time the two operating surgeons need to exchange information regularly. Also, some surgeons will announce transitions between surgical phases to the team:

*For example, we sometimes do one part of arthroscopically and one part open. There you have to say, now we switch to the open procedure* [S03].

Team members may provide information without request or ask questions to support others:

*What I want particularly (…) is that when someone sees a problem, they communicate it. (…) I want him [trainee] to say: Look, I don’t know, is this normal, is this the way it should be, shouldn’t we do it differently. This is very important to me. This is the information I need* [S07].

In case of complications or during technically challenging phases the information exchange was described to be reduced to the necessary minimum and communication becomes more focused and concise:

*If there is a somehow delicate step of the operation (…) where everyone knows now is not the time to ask questions about something else, it takes maximum concentration, and I think in these moments, in these situations, when you really have to focus on the manual step 100% and be totally engaged, then of course, this confines the exchange of information (…) to the actual task, the specific step in the operation* [S14].

Also, in case of intraoperative complications information exchange will aid in diagnosis:

*And along the way, if we have a problem, we must quickly tell anaesthesia. For example, I have recently damaged the thoracic wall (…). They [anaesthetists] told me, something is not as it should be, and I replied, the only thing I can say could have happened on my end could be just that. Then the two of them saw that it all fits and that this was the most likely reason* [S07].

Adequate *situation assessment* is essential to work in a coordinated, goal directed manner. Throughout the procedure team members gain relevant information by constantly monitoring technical equipment and the behaviour of their colleagues (see *team and process monitoring*). This continuous situation assessment serves as a basis for adaptation to situational requirements, including changes in plans and task distribution. Uncoordinated, reactive behaviour was viewed as a common sign for a lack of information and anticipation.

*You can best recognise such things by, well frenetic activity and, yes, again by being reactive, no longer remaining proactive, you can tell when people are not tuned in, do not think ahead* [N04].

Interviewees also pointed out that experiencing flow disruptions, perceiving stress in team members and the feeling of being out of control are indications that the task distribution may be inappropriate and therefore needs to be reconsidered.

*It’s simple. When I realise I’m no longer safe, I don’t have it under control, I can no longer say that he [trainee] is not damaging anything (…) Then you notice something, you have the feeling that he had planned too little, cannot do it manually, that’s the moment where I have to react* [S07].

Participants also described the importance of following the process continuously and closely to anticipate further developments and to adapt to the situational requirements:

*You have to constantly monitor them operating, what they do, and then you see already, aha, now it’s a bit dangerous, now I have to have vascular clamps in theatre. Then I tell my circulating nurse, please go and get them ready, it might be that I need them* [N07].

*Team member information* was mentioned as critical for effective coordination. This includes providing information about oneself and one’s competencies, but also requesting information from specific team members and about their competencies.

*It has happened that I said, look, I’ve never done that before, but I would like to do it, could you stay in theatre with me* [N16].

*Decision making* means to discuss options and resources in order to come to a decision [25]. The interviewed surgeons expressed a tendency for shared decision-making by discussing alternatives in case there are differing opinions within the team of surgeons or in anticipation of unexpected situations during surgery (e.g. complications)*.* Our interviews showed that decision points might be planned for preoperatively. If several surgical alternatives have to be considered, the team will decide on the course of action intraoperatively.

*For example, is the cartilage damaged? Yes / No? How severe is it, how big is it? These are things that you cannot see in advance, not even with imaging methods. Then you plan, there are for example two solutions: a reconstructive method or a stiffening of the joint. These are things that we decide during the surgery. But we plan for this beforehand. (…) There is actually a kind of decision tree already prepared* [S01].

#### Teaching

Although teaching is not a new topic regarding teamwork in surgical settings participants described two different aspects of teaching behaviour. Namely, it includes both guiding a person through a task (e.g. instruct a trainee to perform a surgical step) and educating less experienced team members, trainees or medical/nursing students (e.g. explain steps of the surgical procedure or handling of equipment). Therefore, on the one hand teaching has a coordinating function for the surgical task. On the other hand, it can be seen as a coordination behaviour helping the team to assess the progress of surgery and maintain situation awareness similar to coordination behaviours such as “thinking aloud” or “talking to the room” [34].

Participants mentioned that the consultant surgeon usually discusses the procedure preoperatively with the surgical trainee step-by-step. Surgeons often delegate tasks to the surgical trainee to enable teaching. If the surgeon carries out the surgical procedure, she/he often explains individual steps, tests the trainee’s knowledge by asking questions (e.g. about the anatomical situs), discusses options and corrects the trainee’s mistakes. If a trainee carries out the surgery she/he is being guided through the surgery by the surgeon (depending on education level of the trainee) and is expected to announce her/his further steps.

Nursing trainees receive coaching for a long period of time. Nurses mentioned in the interviews that they are often more cautious when working with nursing trainees so inform them step-by-step about the surgical procedure and the use of instruments and material and anticipate potential mistakes to help prevent them.

*In advance you already know, that there is going to be a trainee that makes mistakes, (…) because it is a learning situation. Then you’re a bit more careful, then you’re thinking ahead, how can we prevent errors. So while I talk more, explain more, inquire if there is ambiguity, maybe I can explain already before we start, how the process might go, or any possible errors or possible ambiguities, or I ask if the trainee has seen something like this before or not. Just a bit cautious and paying more attention* [N14].

Participants mentioned that teaching largely depends on the situation and therefore needs to be adapted to certain circumstances to ensure patient safety suggesting that team members need to balance teaching activities and other surgical tasks. For example, if a procedure is highly demanding for the trainee, teaching might be reduced to providing specific instructions that will allow the trainee to perform the procedure and additional explanations will be provided in a debriefing following the procedure. If the procedure exceeds the trainee’s competence level, the consultant surgeon has to decide whether it is safe and of educational benefit to let the trainee continue under closer guidance or if it is necessary to take over surgery to avert potential damage.

Because time pressure is a common issue in the OR the consultant surgeon may also have to take over if the trainee is not experienced (and fast) enough. While these transitions are mostly described as being initiated by the consultant, surgical as well as nursing trainees are also expected to monitor their own performance and ask for support if they feel unable to manage a certain task, as would be expected from fully qualified clinicians.

#### Leadership

Leadership behaviour has an important coordinative function because it is often focused on effective and efficient accomplishment of the surgical task (i.e. mainly task centric) [35]. Our interviews revealed no clear definition of the perioperative leadership role. Thus, two differentiations have to be kept in mind when discussing this aspect. Firstly, there is a leader, usually the consultant surgeon, who carries the main responsibility for the patient and the surgical procedure (i.e. overall leadership role). Secondly, the person who carries out the surgery (i.e. either the consultant surgeon or a surgical trainee supervised by a consultant) also holds a leadership role (i.e. task centric leadership). Depending on a trainee’s competence level and experience she/ he should be able to lead the team through the surgical procedure and delegate tasks to other OR staff. The team of surgeons decides in advance which surgeon will carry out the procedure (see also planning and general task distribution). According to the interviewees, leadership also depends on certain phases and intraoperative events and thus, may have to be adapted depending on the situation (i.e. transitions in leadership). Interviewees described predefined leadership transitions according to shifting responsibilities for the patient relating to different phases of the surgical process (e.g. anaesthesia team is in charge during transfer to the OR). However, every team member in the OR holds responsibility for her/his tasks and can be considered a leader for her/his work area. In addition, leadership might shift from one team member to another depending on situational demands. For example, in case of anaesthetic problems during surgery, the anaesthetist will take the overall lead, delegate tasks to the surgeons and tell them to stop the procedure if necessary. One nurse described such a situational adaptation in leadership:

*Or even if they say, give me the suture already, we want to close, but the final count has not been completed, we say, stop, no, now we count first, and only then you get it. In these cases it is very clear that you have to seize the leadership role* [N16].

### Situational and contextual drivers

#### Challenging moments

Participants were asked about reasons that may alter the surgical procedure and require a change in coordination. Their answers led to new insights into this topic since they distinguish between two types of challenges that a surgical team may face during the surgical procedure and that have different implications for coordination: *unexpected situations* and *anticipated challenges*.

According to the interviewees, *unexpected situations* can be deteriorating patient condition (e.g. severe blood loss), unexpected or incidental intraoperative findings (e.g. tumor invasion into a blood vessel), iatrogenic injury, procedure more difficult or not feasible as planned, or unavailable instruments. Interviewees mentioned several factors contributing to the occurrence of unexpected situations. Besides patient related (e.g. comorbidities, anti-coagulative medication) and technical reasons (e.g. equipment failure) these factors concerned mainly errors in planning and preparation, underestimation of the situation, team member skills and communication problems between team members.

Interviewees described anticipation and an accurate preoperative planning of the surgical procedure as a crucial task to avoid such unexpected situations. As one surgeon stated:

*When I was very young, I was fascinated by a consultant surgeon that could solve all problems. You know huge problems, a hole cannot be closed, the flap is too short, the screws are wrong, and in the end he could solve everything. With some experience, I noticed that he had produced half the problems himself* [S03].

During surgery the team tries to be prepared as if not everything will go as planned. Unexpected situations can trigger adaptations to the usual coordination behaviours. Communication often decreases in critical situations switching to brief, concise commands. Some surgeons announce critical situations to the whole team and involve them in decision making.

*You have to discuss the issue briefly. Usually we say this is what we have found incidentally, which we did not know of before, or we have this and that complication that we need to take care of now. Then we do it. We discuss these things on the go, so it is rarely the case that one says, stop, we put all the instruments down for a moment and discuss the situation before we continue.* [S07]

If required the team might also pause for a moment to think and discuss how to proceed, what is reasonable and if the required resources are available. Nurses often anticipate future development and organise supplementary instruments and material in advance. Team members also try to minimise distractions:

*These are all relevant things, that the door remains closed, that you know, you need to tell people that no one should come in unless absolutely necessary* [N01].

Due to the possibility of incidental findings surgeons tend to *anticipate challenges* based on the available information and consider alternatives in advance (i.e. develop a plan B, C, D) to reduce the likelihood of unexpected situations. They discuss surgical extensions, potential problems and difficult phases of the surgical procedure within the team of surgeons. Hence, if the primary plan cannot be implemented the team can switch to an alternative approach that has been discussed preoperatively and thus requires minimal additional coordination intraoperatively.

*Because there are many operations where one finds only during surgery, what exactly is going on, how we have to proceed. Often or very often you have a plan, and we know for the most part how the operation will proceed. (…) Then of course the situation unfolds. Or you have incidental findings, which then have some influence. Then you have to change the surgical plan, let’s say, rather than this we use Plan B or Plan C, etc.* [S14].

For certain procedures some surgeons ask clinicians from other specialities to be on standby (e.g. urologist on standby if problems arise with the urethra). Interviewees also mentioned that when working with trainees they always try to ensure there is a backup for them. During difficult surgical steps (e.g. small vessel anastomosis under a microscope) the whole team focuses on this surgically challenging moment, reduces distractions, decreases communication and restricts it to task fulfilment.

#### Climate

While our interview schedule did not contain questions targeting culture or climate within the OR team, participants frequently mentioned the importance of a pleasant *work atmosphere* and a culture that allows team members to speak up.[36] As one surgeon pointed out:

*To create a professional yet relaxed atmosphere where everything revolves around the patient and the operation, (…) and that it is up to me, to ask questions and to expect answers. I think that’s the most important thing* [S14].

The interviewees stated that it is important to establish *open communication* within the whole OR team to prevent communication barriers. Every team member should be able to ask questions or express opinions to other team members regardless of their hierarchical position.

## Discussion

Focusing on surgical teams, this paper describes coordination behaviours and adaptive coordination strategies triggered by situational changes such as non-routine events [20], intraoperative complications and differing level of experience among OR staff. Our findings can be summarised in two main groups: a) the *coordination behaviours and adaptive coordination strategies* and b) *situational and contextual drivers* including the management of *challenging moments* and the supporting effect of a positive *climate* on teamwork.

Even though the interview schedule did not contain any questions regarding climate or culture within the surgical team, participants mentioned the importance of a pleasant work atmosphere and of a communication culture allowing team members to speak up. Their statements suggest that a positive climate is fundamental for a team to be able to adequately coordinate and adapt to varying situational requirements. This finding also shows that we were able to gather rich data material centred around coordination in surgical teams, despite the challenge for the interviewers to extract implicit structures of coordination behaviour in surgical teams. Participants often found it difficult to verbalise the cognitive processes underlying their coordination behaviour which they perceive to be performed “automatically” [24,37]. Also, while we explicitly asked interviewees to refer to perioperative phases of surgical procedures they frequently made reference to the preoperative phase, especially when talking about planning or task distribution. The high level of coordination beforehand and its importance was also reflected in statements by the interviewees that everything has to be “set” and “clear” for the intraoperative phase. Therefore, when investigating coordination in surgical teams it is important to consider the different phases in which certain coordination activities usually are performed and the interdependencies of coordination activities across phases.

This “pre-process coordination” in form of preoperative planning and task distribution can also be seen as a strategy of using low workload phases to prepare for higher workload phases [38]. At this stage, the team aims to establish a shared mental model [30,31] to enable synchronised performance of the whole team. Anticipating the further proceeding, identifying other team member’s needs and adapting one’s own behaviour to changing situational requirements are extremely difficult unless the team possesses valid shared mental models [39]. Interviewees also mentioned that the standardised use of the preoperative safe surgery checklist positively impacts on coordination in the surgical team, by providing an opportunity to update the team’s mental model. Previous research highlights that the use of the safe surgery checklist has shown improvements in surgical outcomes [33] as well as in safety climate, teamwork and communication [40,41].

In addition to the value of these pre-procedural strategies for facilitating coordination, our results highlight the dynamic nature of surgical teamwork and the adaptations necessary to ensure optimal coordination (e.g. information exchange may vary according to occurrences during surgery). This becomes particularly apparent in situations that are challenging for the team (e.g. unexpected or more complex). In these situations, communication was described to decrease and become more focused and concise.

Furthermore, participants highlighted the importance of constantly monitoring other team members’ activities and of the surgery’s proceedings for creating and maintaining situation awareness. Timely and accurate situation assessment is a central input to decision making in surgical teams [42] and to the correct prioritisation of tasks.

Our study provides ample evidence for adaptive coordination in surgical teams. This adaptation to situational requirements can be seen as a strategy to prevent potential problems and critical incidents. Former research has shown that engaging in preventable minor problems enhances the ability of surgical teams to deal with unpreventable major problems [4]. Concerning challenging moments, our interviewees mentioned anticipatory adaptations to difficult phases of the surgery and to anticipated problems by developing alternative plans that can aid in intraoperative coordination. In addition, interviewees mentioned coordination strategies aimed at managing unexpected situations should they occur (e.g. minimise distractions). Previous research described such transitions from routine to more effortful functioning in terms of “slowing down” [43,44]. These slowing down moments can either be planned (i.e. anticipating critical points and intentionally transitioning from routine to more effortful) or unplanned (i.e. situational responding to unexpected situations). Interestingly the “slowing down” phenomenon was mentioned in our interviews although we did not specifically focus on this issue.

Leadership constitutes a special aspect of coordination. Our interviewees mentioned that the overall leadership role is usually held by the consultant surgeon. Nevertheless, every team member was thought to have responsibility for her/his own work area and hence, holds a leadership role. Previous research also points out that leadership can be shown by several team members [45,46]. Yun et al. [47] discuss the “importance of leader adaptability”. They make two differentiations: one is to change the leader (“between-leader approach”), the other is that one leader changes her/his way of leading (e.g. directive vs. empowering) to adapt to situational changes (“within-leader approach”). The “between-leader approach” was also apparent in our interviews. Interestingly, there appeared to be a tight link between leadership and teaching in the sense of a supervisory role carried by the consultant surgeon. Our interviewees described a rather implicit process of intraoperative negotiation and decision making. How much control the surgeon is willing to give to the trainee depends on occurrences during surgery and the surgeon’s judgement of the trainee’s competencies. Moulton et al. [48] also highlight this “control dynamic” between the consultant surgeon and the trainee. They report how surgeons balance the responsibilities for patient safety and teaching of surgical trainees by illustrating several strategies for negotiating manual control. Balancing the dual responsibility for patient safety on the one hand and providing teaching opportunities on the other is an important issue in the teaching hospital setting. Compared to previous research on anaesthesia teams the surgical team seems to be more complex due to the consolidation of two sub-teams (i.e. surgeons and nurses) into one. This makes leadership and team management even bigger challenges.

### Limitations

There are several limitations to this study. Firstly, talking about coordination behaviours means to talk about processes that are often perceived as occurring automatically. Interviewees had difficulties with verbalising these coordination behaviours explicitly. We overcame this challenge by asking probing questions and by inviting the participants to provide examples and refer to their prior experiences. Secondly, the labels we used in describing the coordination behaviours and adaptive coordination strategies appear to have a considerable overlap with the behavioural markers for non-technical skills in surgical teams, which were already explored in previous research [23,25,49,50]. These systems provide behavioural markers for good performance and therefore enable an assessment of the team’s performance. Our aim, based on this interview study, is the development of a system that enables a descriptive, structured observation of surgical teams and their coordination behaviour. Such a system will provide team process descriptions that can be linked to performance data in order to identify effective coordination behaviours. Thirdly, during the interviews we noticed that there is no consistent understanding regarding the leadership role. Yet this brought us to the conclusion that leadership is variable in nature, depending on situational requirements and with the ability to change through different phases of the surgical procedure. Fourthly, our study was limited to Switzerland and future cross-cultural research is needed to explore the generalizability of our results. Fifthly, this study focuses on the surgical team defined as consisting of surgeons and theatre nurses. Thus, other members of the OR team such as the anaesthesia sub-team were not interviewed despite their interactions with the surgical team during surgery. Sixthly, our analysis of the data material had no focus on professional differences between nurses and surgeons. Although, this might be an interesting point our priority was to gather relevant information that stands for the whole team. Finally, our interviews focused on eliciting the subjective views of individual clinicians. Other research approaches such as focus group studies might offer complementary information.

## Conclusions

The present study is complementary to previous research on non-technical skills and extends the research on adaptive coordination in the OR by focusing on surgical teams. Focusing on clinicians’ subjective views it highlights the central role of coordination and describes coordination behaviours and adaptive coordination strategies employed by surgical team members to ensure effective team performance. Furthermore, situational characteristics are described which may influence team coordination and thus, require the team to behave adaptively.

Based on these findings our next step is to develop a structured observation system specific to coordination processes in surgical teams and to apply it in observational research. We hope that these insights to adaptive coordination strategies used by surgical teams will allow for a better understanding of teamwork in this particular setting and thus help to improve teamwork and patient safety. Another interesting point for the management of a surgical department is the supporting effect of a positive climate and pre-process coordination in form of preoperative planning. It should be a managerial responsibility to a) create a culture that enables every team member to speak up and voice their concerns and b) design the process of preoperative planning so that every surgical team member is involved appropriately.

## Additional file

Additional file 1:
**Interview schedule.**
