# Supplementary material for: Adaptive coordination in surgical teams: an interview study
Source: BMC Health Serv Res. 2015 Apr 1;15:128. doi: 10.1186/s12913-015-0792-5 (PMC4389413; doi:10.1186/s12913-015-0792-5)
Supplement: Additional file 1: — Interview schedule. [file 12913_2015_792_MOESM1_ESM.docx]

**Additional file 1: Interview schedule**

| From your point of view, what constitutes good teamwork? |
| --- |
| Do you usually discuss the team members’ roles prior to surgery? |
| What might cause a deviation from the way you normally do things?  How is this decision made? |
| How should the tasks be distributed between consultant, trainee, scrub nurse and circulator? |
| Who defines the task distribution and on what basis? |
| Are you involved in teaching? If yes, how do you integrate this task with other tasks you have to fulfil during surgery? |
| How do you gain information if the task distribution is adequate for the situation at hand? |
| What could cause an adaptation in task distribution? |
| Who is the leader of the overall theatre team? |
| How is this defined? |
| Are there situations that require a change in leadership? |
| How is the decision for such a change derived and put into practice? |
| When do you need information from other surgical team members? What information do you need? |
| When do you have to pass information to other team members? What information do you have to pass? |
| How do you know about the information needs of the other team members? |
| Does this information exchange always look the same or are there situations/ incidents that might change information needs? |
| Nurses:  When do you have to coordinate with the anaesthesia team? Why?  When do you have to coordinate with the surgeons? Why? |
| Surgeons:  When do you have to coordinate with the nursing team? Why?  When do you have to coordinate with the anaesthesia team? Why? |
| Are there certain points during surgery when the whole OR team needs to coordinate among each other? |
| Are there certain phases during which you monitor the work process of others or their communication more closely? To get what information? |
| In which situations do you need assistance from other team members? In which situations do you need to offer assistance to others? |
| Do you have any additional comments? |
